# Anti-inflammatory and anti-proliferative effect of herbal medicines (APR) in RAW264.7 cells

**DOI:** 10.3892/mmr.2014.2033

**Published:** 2014-03-10

**Authors:** HAN-SEOK CHOI, HYE SOOK SEO, SOON RE KIM, YOUN KYUNG CHOI, YONG-CHEOL SHIN, SEONG-GYU KO

**Affiliations:** Laboratory of Clinical Biology and Pharmacogenomics and Center for Clinical Research and Genomics, Institute of Oriental Medicine, Kyung Hee University, Seoul 130-701, Republic of Korea

**Keywords:** *Angelica gigas Nakai*, *Panax ginseng*, *Rhus verniciflua Stokes*, inflammation, RAW264.7, lipopolysaccharide, reactive oxygen species

## Abstract

The objective of the present study was to analyze the effect of a mixture of medicinal plants [*Angelica gigas Nakai*, *Panax ginseng* and *Rhus verniciflua Stokes* (APR)] on lipopolysaccharide (LPS)-induced inflammatory responses in the murine macrophage cell line RAW264.7. Cells were treated with APR and LPS at various concentrations and indicated times. WST assay, trypan blue assay and quantification of activated cells demonstrated that APR suppressed cell proliferation in a dose-dependent manner. APR induced G1 cell cycle arrest and inhibited the LPS-induced phosphorylation of protein kinase B (AKT), extracellular signal-regulated kinase (ERK), mitogen-activated protein kinase (p38) and necrosis factor κB (NF-κB). APR also suppressed nitric oxide synthase isoform (iNOS) and prostaglandin endoperoxide synthase 2 (Cox-2) messenger ribonucleic acid (mRNA) expression induced by LPS. Furthermore, APR decreased LPS-induced intracellular reactive oxygen species (ROS) levels, mitochondrial membrane potential, as well as induced PARP and caspase-3 cleavage, suggesting that APR causes apoptosis. In conclusion, the present study indicated that APR may be advantageous in treating inflammatory disease.

## Introduction

Inflammatory response is a major defense mechanism against pathogens and chemical or mechanical injury. These phenomena are mediated by inflammatory cells including macrophages. Activated macrophages produce reactive oxygen species (ROS) and nitric oxide (NO), and cause substantial oxidant injury to surrounding tissue (1–3). Extensive laboratory and clinical evidence indicates that chronic inflammation contributes to cancer (4). Oxidative stress-induced neuron injury induces a variety of neurodegenerative diseases including Alzheimer’s and Parkinson’s disease as well as cerebral ischemia (5).

The dried roots of *Angelica gigas Nakai* (AGN) have been traditionally used in Oriental Medicine. It is known in Korean as ‘Cham-dang-gui’. Several coumarin derivatives and a pectic polysaccharide were isolated from AGN (6). These derivatives are known to inhibit cancer cell adhesion and invasion (7), have anti-diabetic activity (8), suppress androgen-induced and -independent cell proliferation, and cause anti-inflammatory activities (9) and neuroprotective activity (10).

*Panax ginseng* (PG), one of the most well-known herbal medicines, has been commonly used in East Asia. Total saponins and ginsenosides are the major active components of PG (11). Ginseng has various biological activities, including inhibition of tumor-induced angiogenesis, prevention of tumor invasion and metastasis (12), as well as anti-infective (13), anti-diabetic (14), anti-inflammatory (15), and neuroprotective activities (16).

*Rhus verniciflua Stokes* (RVS) has traditionally been used as an ingredient in East Asian Medicine for the treatment of gastritis, stomach cancer and atherosclerosis. The compounds identified from RVS are as follows: Gallic acid, protocatechuic acid, quercetin, fustin, fisetin, sulfuretin and butein (17). RVS protects from oxidative damage by scavenging reactive oxygen species (ROS) (18) and it has anti-proliferative, anti-cancer and anti-inflammatory effects (19).

In the present study, the effect of a mixture of three medicinal plants AGN, PG*,* RVS (APR) on lipopolysaccharide (LPS)-induced inflammatory responses in the mouse macrophage cell line RAW264.7 was evaluated. It was assessed whether an ethanolic (EtOH) extract of APR suppresses LPS-induced inflammatory responses in RAW264.7. The present study also investigated whether APR exhibits anti-proliferative activity regulating intracellular molecules associated with cell survival and apoptosis.

## Materials and methods

### Cell culture

RAW264.7 mouse macrophage cells were obtained from the Korea Cell Line Bank (Seoul, Korea). Cells were cultured in Dulbecco’s modified Eagle’s medium (DMEM) supplemented with 10% heat-inactivated fetal bovine serum (FBS) and 1% antibiotics (penicillin-streptomycin) at 37°C in a 5% CO_2_ humidified incubator.

### Extraction of medicinal plants (APR)

Medicinal plants used in the present study were purchased from Omniherb (Daekoo, Gyeongsangbuk-do, Korea). The powder with a mass of 100 g (AGN and PG, root and RVS, bark) was extracted twice with 80% (v/v) ethanol (Duksan Pharmaceutical Co., Ltd., Ansan, Republic of Korea) by using an Ultra-sonicator (Branson, Danbury, CT, USA) for 30 min at room temperature. The alcoholic extract was filtered through a 0.22 μm filter, the solvent was evaporated at 40°C and the residue freeze-dried. The yields of the extracts were 38.2, 26.4 and 13.7% (w/w) for AGN, PG and RVS, respectively. The plant extract mixture was prepared as AGN : PG : RVS = 1:1:0.1.

### Cell proliferation assay

The cell proliferation rate was determined using the water soluble tetrazolium (WST) assay following treatment with APR. The WST assay is based on the cleavage of the yellow tetrazolium salt to purple formazan crystals by metabolically active live cells.

RAW264.7 cells (1×10^4^ cells/well) were seeded in 96-well plates, incubated overnight and treated with APR. Following 24 h of incubation, 10 μl WST solution was added to 100 μl cell culture medium and the plates were incubated for a further 2 h. The optical density was determined at 490 nm using an ELISA reader (Molecular Devices, Palo Alto, CA, USA).

### Cell death assay

Cell death following APR treatment was determined using the trypan blue assay. Trypan blue selectively stains dead cells. RAW264.7 cells were treated with APR for 12 and 24 h, respectively. The cells were then suspended and stained with trypan blue solution (Sigma Aldrich; St. Louis, MO, USA). The cell number was determined by counting using a hemocytometer.

### Cell surface observation

Cells were seeded into 60-mm culture dishes at a density of 3×10^5^ cells/dish. The following day, the cells were treated with APR for 12 h. The cell surface was observed by capturing an image using a camera (Olympus) attached to a microscope.

### Mitochondrial membrane potential analysis

The loss of mitochondrial membrane potential is a specific characteristic of apoptosis. 5,5′,6,6′-Tetrachloro-1,1′,3,3′-tetraethylbenzimidazolylcarbocyanine iodide (JC-1) is a membrane-permeable dye widely used for determining the mitochondrial membrane potential using flow cytometry and fluorescent microscopy. Cells were seeded into 60 mm culture dishes at a density of 3×10^5^ cells/dish. The following day, the cells were treated with APR for 24 h. Cells were harvested from each culture dish, washed with phosphate-buffered saline (PBS), suspended in PBS containing 2 μM JC-1 and incubated for 30 min at 37°C in the dark. The data were analyzed by FACSCalibur flow cytometry (BD Biosciences, Franklin Lakes, NJ, USA).

### Assessment of intracellular reactive oxygen species (ROS) levels

The molecule 2′,7′-dichlorofluorescein diacetate (DCFH-DA) freely permeates cells, and following incorporation into cells, is converted into the fluorescent 2,7-dichlorofluorescein (DCF) by oxidative substances, revealing the intracellular production of redox-active substances. It has been widely used to investigate oxidative damage in intact cells. Cells were seeded into 35 mm culture dishes containing glass coverslips. Following different pretreatments, the cells were washed with PBS and incubated with 20 μM DCFH-DA for 30 min at 37°C in the dark. Following washing with cold PBS, the fluorescence was captured using a laser confocal scanning microscope (LSM 510; Carl Zeiss, Jena, Germany) and a FACSCalibur flow cytometer (BD Biosciences, San Jose, CA, USA). DCF fluorescence was measured at an excitation wavelength of 488 nm and emission at 515–540 nm.

### RNA extraction and reverse transcription polymerase chain reaction (RT-PCR)

Cells were collected by centrifugation and RNA was extracted using an Invitrogen kit (Grand Island, NY, USA), according to the manufacturer’s instructions. Primers were designed for RT-PCR for nitric oxide synthase isoform (iNOS) and prostaglandin endoperoxide synthase 2 (Cox-2). The primer sequences were as follows: (sense) 5′-GGAGAGACTATCAAGATAGT-3′ and (antisense) 5′-ATGGTCAGTAGACTTTTACA-3′ for COX-2; (sense) 5′-AATGGCAACATCAGGTCGGCCATCACT-3′ and (antisense) 5′-GCTGTGTGTCACAGAAGTCTCGAACTC-3′ for iNOS; (sense) 5′-GAGGGGCCATCCACAGTCTTC-3′ and (antisense) 5′-CATCACCATCTTCCAGGAGCG-3′ for glyceraldehyde 3-phosphate dehydrogenase (GAPDH). The sequencing involved 30 cycles with denaturation at 94°C for 45 sec, annealing at 55°C for 45 sec and extension at 72°C for 45 sec. The resulting PCR products were resolved on 1% agarose gels containing ethidium bromide.

### Western blot analysis

Whole cell lysates from cells treated with DMSO and APR in the presence or absence of LPS were prepared by washing with ice-cold PBS and lysis with a radioimmunoprecipitation assay (RIPA) buffer. Equal amounts of protein (30 μg) from the cell lysates were boiled for 5 min in SDS-PAGE sample buffer, resolved by 10% SDS-PAGE, transferred to nitrocellulose membranes at 80 V for 1.5 h and visualized using western blot analysis and chemiluminescence.

## Results

### Effects of APR on cell viability

**Figure 1:**
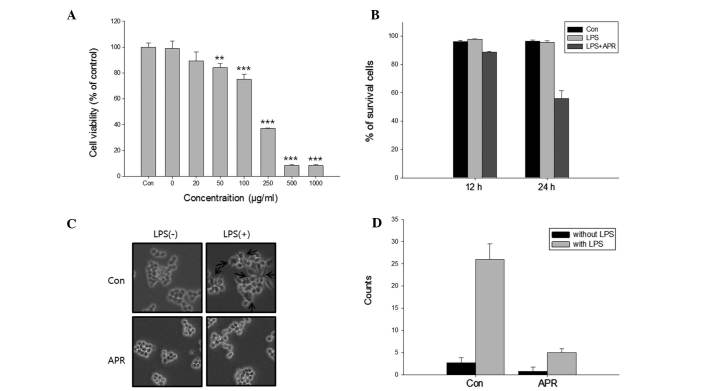
Effect of APR on proliferation and death of RAW264.7 cells. (A) Cell proliferation rate was determined using the WST assay. RAW264.7 cells were treated with APR at concentrations of 0–1,000 μg/ml in the absence or presence of 1 μg/ml LPS for 24 h. APR significantly inhibited cell proliferation at concentrations of 250–1,000 μg/ml. Each value is the mean ± standard deviation (^**^P<0.01; ^***^P<0.001 according to the Student’s t-test). (B) The cell death rate was determined using trypan blue assay. RAW264.7 cells were treated with 150 μg/ml APR in the absence or presence of 1 μg/ml LPS for 12 and 24 h, respectively. APR caused cell death in a time-dependent manner. (C) LPS-induced morphological changes were reversed by APR in RAW264.7 cells. Cells were treated with APR in the absence or presence of 1 μg/ml LPS for 12 h. APR inhibited the activation of RAW264.7 cells induced by LPS. (D) Number of cell surface changes in RAW264.7 cells. Cells were treated with APR in the absence or presence of 1 μg/ml LPS for 12 h. The cell surface was observed on an image captured by a camera attached to a microscope. APR, *Angelica gigas Nakai*, *Panax ginseng* and *Rhus verniciflua Stokes*; LPS, liposaccharide.

The anti-proliferative effects of APR were assessed on RAW264.7 mouse macrophage cells. First, the inhibitory effect of APR on cell proliferation was investigated using the WST assay (Fig. 1a). For this purpose, the cells were treated with APR at concentrations of 0–1,000 μg/ml and 1 μg/ml LPS for 12 h. LPS alone did not show any proliferative activity in RAW264.7 cells. However, APR significantly inhibited cell proliferation at concentrations of 250–1,000 μg/ml, suggesting that APR inhibits the growth of RAW264.7 cells. Second, the cell death rate was determined using the trypan blue assay during APR treatment (Fig. 1b). At 24 h of incubation, APR significantly decreased the percentage of surviving cells.

Changes in the cellular morphology under LPS and APR treatment were also observed (Fig. 1c). Untreated RAW264.7 cells are circular shaped. However, following incubation with LPS, the cells exerted an anomalous shape and became elongated. Microscopic examination of cells revealed a reversal of LPS-induced alteration in cell morphology following treatment with APR.

On the other hand, Fig. 1d shows the number of cell surface changes following LPS and/or APR treatment in RAW264.7 cells. APR significantly decreased the number of cell surface changes induced by LPS. These results suggest that APR inhibits the proliferation of RAW264.7 cells and blocks the LPS-induced activation of RAW264.7 cells.

### APR decreases iNOS and COX-2 mRNA expression in RAW264.7 cells

**Figure 2:**
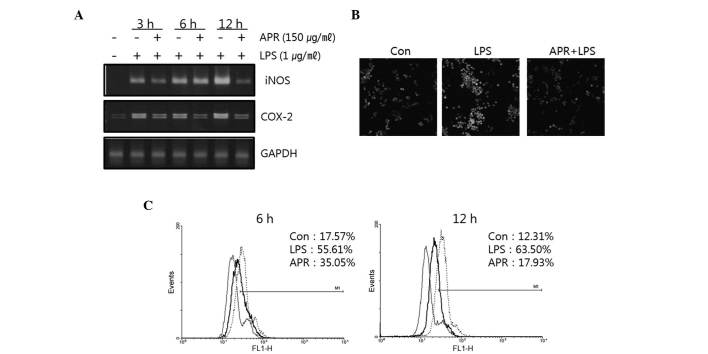
APR decreased iNOS and Cox-2 mRNA expression and suppressed intracellular ROS levels induced by LPS in RAW264.7 cells. (A) The expression of iNOS and COX-2 mRNA was assayed by RT-PCR. Cells were treated with 150 μg/ml APR in the absence or presence of 1 μg/ml LPS for 12 h. APR suppressed iNOS and COX-2 mRNA expression induced by LPS. ROS were detected by (B) laser confocal scanning microscopy and (C) flow cytometry with DCFH-DA. Cells were treated with 150 μg/ml APR in the absence or presence of 1 μg/ml LPS for 6 h and incubated with DCFH-DA for 30 min. DCF fluorescence was measured using a confocal laser-scanning microscopy and FACSCalibur. APR inhibited ROS generation induced by LPS. APR, *Angelica gigas Nakai*, *Panax ginseng* and *Rhus verniciflua Stokes;* iNOS, nitric oxide synthase isoform; Cox-2, prostaglandin endoperoxide synthase 2; ROS, reactive oxygen species; LPS, liposaccharide RT-PCR, reverse transcription polymerase chain reaction; DCFH-DA, 2′,7′-dichlorofluorescein diacetate; GADPH, glyceraldehyde 3-phosphate dehydrogenase.

Since iNOS and ROS are mediators in inflammatory reactions, the expression of iNOS mRNA and COX-2 mRNA in RAW264.7 cells was assessed. APR suppressed iNOS mRNA and COX-2 mRNA expression induced by LPS in RAW264.7 cells (Fig. 2a), suggesting that APR suppresses inflammatory reactions.

### APR decreases ROS levels in RAW264.7 cells

ROS levels were measured by confocal microscopy (Fig. 2b) and fluorescence-activated cell sorting (FACS) analysis (Fig. 2c) using DCFH-DA. Following LPS treatment cellular ROS levels were increased. However, APR co-treatment inhibited ROS generation induced by LPS in a time-dependent manner.

### APR affects cell cycle

**Figure 3:**
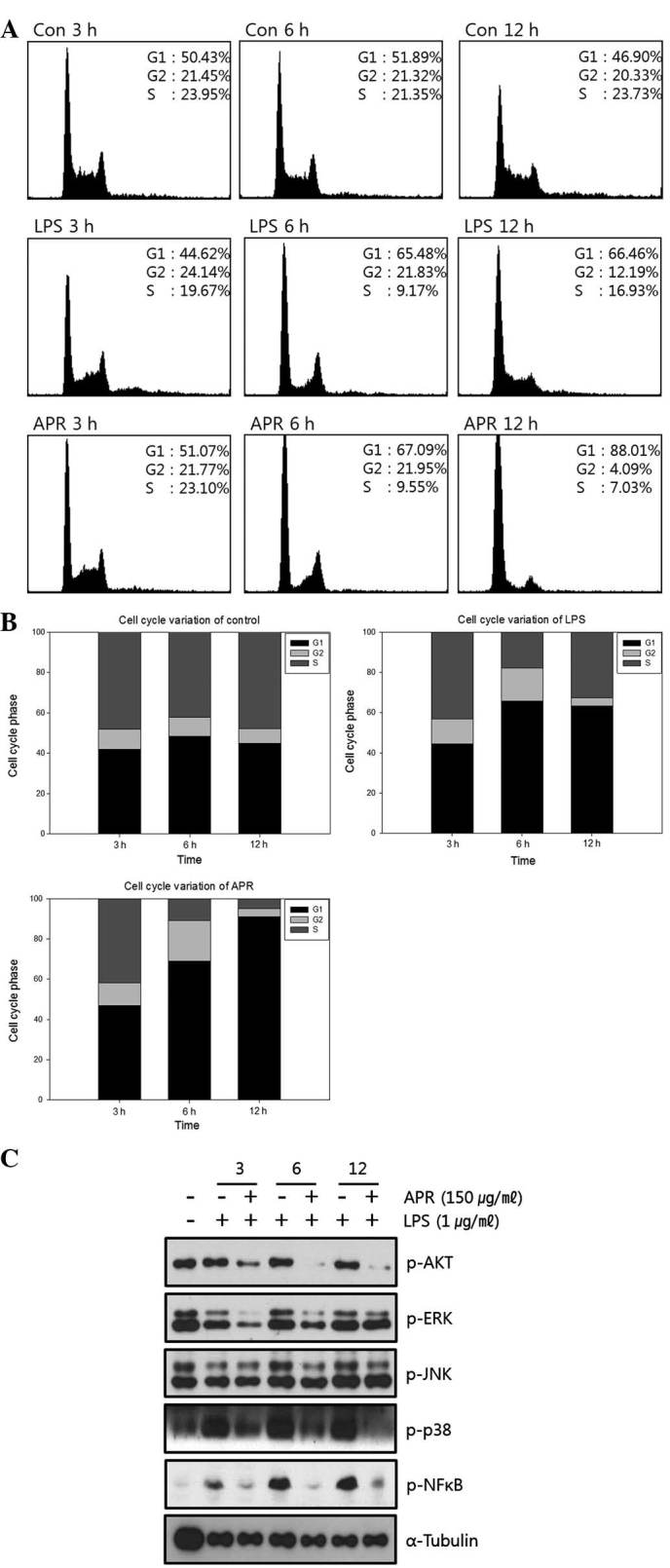
APR affects cell cycle. (A) Cell cycle was analyzed using FACS. Cells were treated with APR (150 μg/ml) in the absence or presence of 1 μg/ml LPS for 3, 6 and 12 h, respectively. Cells were stained with propidium iodide solution and analyzed by flow cytometry. APR caused G1 arrest. (B) G1, S and G2/M phase fractions were quantified from DNA histograms data in A. (C) Effect of APR on the expression of intracellular molecules in RAW264.7 cells. Cells were treated with APR (150 μg/ml) in the absence or presence of 1 μg/ml LPS for 3, 6 and 12 h, respectively. Cell extracts were subjected to western blot analysis with specific antibodies. APR had inhibitory effects on AKT, ERK, p38 and NF-κB phosphorylation. APR, *Angelica gigas Nakai*, *Panax ginseng* and *Rhus verniciflua Stokes;* FACS, fluorescence-activated cell sorting; LPS, liposaccharide; AKT, protein kinase B; ERK, extracellular signal-regulated kinase; p-JNK, c-Jun terminal kinase; p38, p38 mitogen-activated protein kinase; NF-κB, necrosis factor κB.

The effect of APR on the cell cycle was assessed using FACS analysis. As shown in Fig. 3a and b, APR caused G1 phase arrest at 6 and 12 h of incubation in a time-dependent manner. Fig. 3c shows the expression of intracellular molecules associated with cell proliferation, assessed using western blot analysis.

### APR suppresses protein kinase B (p-AKT) and extracellular signal-regulated kinase (p-ERK) expression induced by LPS in a time-dependent manner

c-Jun terminal kinase (JNK) phosphorylation was not suppressed by APR, but p38 mitogen-activated protein kinases (p38) phosphorylation induced by LPS was downregulated in a time-dependent manner by APR. Necrosis factor κB (NF-κB) phosphorylation induced by LPS was inhibited by APR. These results suggest that APR inhibits cell proliferation by suppression of the phosphorylation of AKT, ERK, p38 and NF-κB.

### APR induces apoptosis through the mitochondrial death pathway

**Figure 4:**
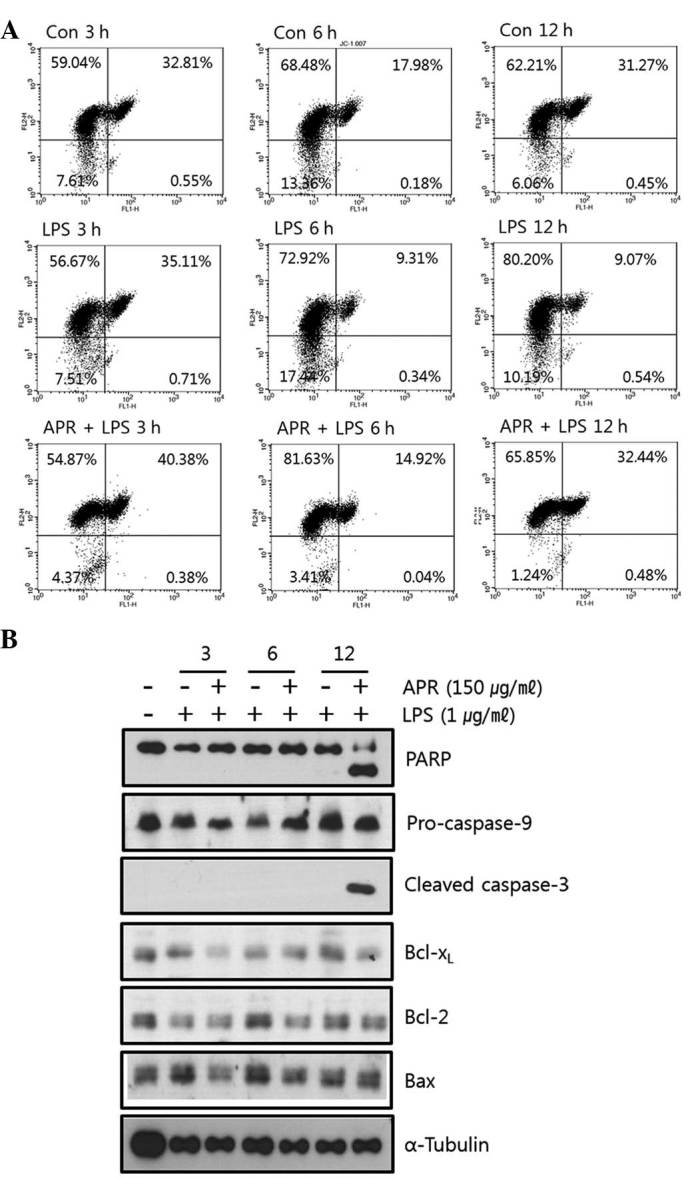
APR induces apoptosis through mitochondrial death pathway. (a) Cells were treated with APR (150 μg/ml) in the absence or presence of 1 μg/ml LPS for 3, 6 and 12 h, respectively. Following incubation with JC-1, cells were analyzed by FACS. APR decreased the mitochondrial membrane potential. (b) Effect of APR on caspase activity and apoptosis in RAW264.7 cells. Cells were treated with APR (150 μg/ml) in the absence or presence of 1 μg/ml LPS for 3, 6 and 12 h, respectively. Cell extracts were subjected to western blot analysis with specific antibodies. APR cleaved PARP and caspase-3. APR, *Angelica gigas Nakai*, *Panax ginseng* and *Rhus verniciflua Stokes*; LPS, liposaccharide; JC-1, 5,5′,6,6′-tetrachloro-1,1′,3,3′-tetraethylbenzimidazolylcarbocyanine iodide; FACS, fluorescence-activated cell sorting; PARP, poly adenosine diphosphate ribose polymerase; Bcl-x_L_, B-cell lymphoma-extra large protein; Bcl-2, B-cell lymphoma 2; Bax, BCl-2-associated protein x.

The loss of mitochondrial membrane potential (ΔΨ) is a hallmark of apoptosis. Mitochondrial permeability transition is an important step in the induction of cell apoptosis. JC-1 is able to selectively enter mitochondria and reversibly change the color from red to green as the membrane potential decreases. Thus, cells incubated with LPS and/or APR were stained with JC-1 and assessed through FACS analysis. It was found that APR decreased the mitochondrial membrane potential in RAW264.7 cells as shown in Fig. 4a. Treatment with LPS alone resulted in a more stable mitochondrial membrane potential (green fluorescence, 9.61% at 12 h) compared with the control (green fluorescence, 31.72% at 12 h). APR perturbed this stability induced by LPS and decreased the mitochondrial membrane potential (green fluorescence, 32.92% at 12 h), suggesting that APR induces apoptosis through the mitochondrial death pathway.

It was also confirmed whether APR regulated the expression of apoptosis-associated molecules. As shown in Fig. 4b, APR induced the cleavage of poly adenosine diphosphate ribose polymerase (PARP) and caspase-3 which are apoptotic products, confirming that APR causes apoptosis.

## Discussion

The present study revealed that an ethanolic extract of APR suppressed the LPS-induced inflammatory responses in the mouse macrophage cell line RAW264.7.

Inflammation is a host’s protection method against pathogens and is stimulated by diverse microbial products (20). Pro-inflammatory cytokines may aggravate the severity of multiple inflammatory diseases (21). Diverse inflammatory agents are able to activate NF-κB. Activation of NF-κB induces inflammation and increases cell survival and tumor cell transformation (22). Mitogen-activated protein kinase (MAPK) pathways are also associated with inflammation. The ERK pathway is activated by inflammation (23).

APR effectively inhibited the growth stimulation and activation of RAW264.7 cells induced by LPS. APR significantly inhibited cell growth at concentrations of 250–1,000 μg/ml and induced cell death at 150 μg/ml (24 h). It was shown that APR negated the morphological changes of RAW264.7 cells induced by LPS. APR decreased intracellular ROS levels and suppressed iNOS and Cox-2 mRNA expression induced by LPS. APR decreased the mitochondrial membrane potential and cleaved caspase-3 and PARP. This result indicates that APR inhibits inflammation and induces apoptosis through mitochondrial death pathways.

Since APR has an anti-inflammatory effect, it may be used for the treatment of inflammatory diseases, including rheumatoid arthritis and asthma (24). Transformation of a normal cell into tumor cell is closely associated with chronic inflammation (25), and accordingly, APR (AGN, PG and RVS) may be a useful compound for cancer prevention.
